# The Impact of Cybervictimization on Psychological Adjustment in Adolescence: Analyzing the Role of Emotional Intelligence

**DOI:** 10.3390/ijerph17103693

**Published:** 2020-05-23

**Authors:** Jesús F. Estévez, Elizabeth Cañas, Estefanía Estévez

**Affiliations:** Department of Health Psychology, Universidad Miguel Hernández de Elche, 03202 Elche, Spain; jesus.estevez01@goumh.umh.es (J.F.E.); ecanas@goumh.umh.es (E.C.)

**Keywords:** cybervictimization, self-concept, depression, life satisfaction, emotional intelligence

## Abstract

Cybervictimization has been associated with serious emotional adjustment problems such as low self-concept and depressive symptomatology. In addition, these problems can negatively affect the well-being of the victims, manifesting in their levels of satisfaction with life. However, it should be noted that not all cybervictims develop these consequences with the same intensity. These differences seem to be related to the development of emotional intelligence (EI), as it can positively influence adolescents’ emotional adjustment and well-being even when problems arise. The objective of this work was to analyze the role of EI on cybervictimization and adolescents’ emotional adjustment, especially in self-concept, depression, and life satisfaction. The participants in the study were 1318 adolescents of both sexes and aged between 11 and 18 years (*M* = 13.8, *SD* = 1.32), from four secondary compulsory education centers in Spain. EI influences the relationship between self-concept and life satisfaction, and between depression and life satisfaction. In addition, the relationships of cybervictimization with self-concept and depression are influenced when introducing EI and its dimensions (emotional attention, clarity, regulation). These data support the idea that EI may affect the relationship between cybervictimization and adolescents’ emotional adjustment.

## 1. Introduction

The increasingly digital world has expanded the use of information and communication technologies (ICTs) as a way for social interactions among children and adolescents, creating new opportunities for cyberbullying [1]. Cyberbullying involves the use of ICTs to carry out a series of direct (occurring in the private domain) and indirect (occurring in a public area of cyberspace) acts, intended to harm another (the victim) who cannot easily defend themselves [2]. This behavior identifies some fundamental aspects such as repetition because posting contents online in itself constitutes repetition as they can be viewed and forwarded repeatedly; intentionality of the act, which is related to the repetition; power imbalance, that is the perpetrator’s perceived and actual power over the victim related to controlling material in cyberspace [2,3]; and anonymity and publicity, which occur when the victim does not know the bully’s identity and a large audience is involved via cyberspace [4].

The characteristics of this behavior have been associated with several adverse consequences for the victims, especially concerning the emotional adjustment of adolescents [1,5]. Among the effects of cybervictimization, considerable research has shown that cybervictims report higher levels of depressive symptomatology [6,7]. The characteristics of cybervictimization, such as the repetitiveness of the acts, the anonymity of the cyberaggressor, and the wide audience, among others, may increase the risk of suffering from depressive symptomatology in cybervictims [8]. Cybervictims also tend to show a negative global self-concept and particularly negative self-evaluations in some of the personal dimensions [9,10]. Thus, from a multidimensional perspective of self-evaluation, previous studies with cybervictims have suggested that online victimization may damage academic performance and self-concept [11], family climate and child–parent relationships [12], and the general self-confidence [13] of those who suffer this problem.

Cybervictimization has also been associated with life satisfaction [14]. Life satisfaction, considered in the scientific literature as an important indicator of subjective well-being, refers to a cognitive, judgmental process, which depends upon a comparison of one’s circumstances with what is thought to be an appropriate standard. Additionally, the judgment of how satisfied people are with their present state of affairs is based on a comparison with a standard which each individual sets for themselves; it is not externally imposed [15]. Recent studies have revealed that cybervictims show a lower level of life satisfaction in comparison to those students not involved in victimization situations [9,12]. Other researchers have suggested that online perpetration causes hopelessness and discomfort in the victims, which have in turn been negatively associated with some domains of adolescents’ subjective well-being, specifically life satisfaction [16]. These findings suggest that cyberbullying is a severe problem that seriously affects the emotional and psychosocial well-being of the involved adolescents.

On another hand, it should be noted that not all cybervictims develop the same negative consequences or with the same degree of intensity [17,18]. It has been suggested that the different impact cybervictimization has on the victims could be explained through certain variables, especially related to emotional skills, which may act as protective factors [19]. According to recent studies, one of the focus variables may be Emotional Intelligence (EI), which has been identified as a potent construct for the protection and promotion of adolescents’ emotional adjustment and well-being [20,21].

EI is the ability to perceive, assimilate, understand, regulate emotions [22], as well as, to recognize one’s own emotions and those of others, to motivate oneself, and to manage emotions both about oneself and others [23]. The model proposed by Mayer and Salovey [22] highlights three components of EI: (1) Attention—ability to perceive emotions of the self and the others; (2) Clarity—ability to understand emotional information, how emotions combine and change over time, and the ability to appreciate emotional meanings; and (3) Regulation—to remain open to feelings and to monitor and regulate emotions to promote understanding and personal growth. These three components or dimensions have been studied in the context of victimization in adolescence [24], indicating that, in general, high levels of EI are negatively associated with cybervictimization in adolescents [19,24].

More specifically, studies about the multidimensional analysis of EI in cases of cybervictimization have reported that cybervictims pay considerable attention to their own emotions (emotional attention), but have difficulties to understand (emotional clarity) and manage (emotional regulation) them [25,26]. In line with these results, Estévez et al. [26] indicated that cybervictims’ emotional imbalance prevents them from concentrating on choosing and using adaptive coping strategies and recovering emotional balance and life satisfaction. This convergence with investigations that indicate that the development of typical EI emotional skills can reduce the risk in adolescent cybervictims of eventually developing psychosocial problems as a consequence of cyberbullying [19,27].

Although these results suggest that EI could influence the relationship between cybervictimization and emotional adjustment, studies on this topic are limited. However, the scant results available indicate that high levels of EI could be very beneficial for cybervictims, as this may prevent psychosocial and emotional adjustment problems [21]. In this sense, Elipe et al. [18] found that cybervictims with high levels of emotional attention and regulation reported lower levels of anger and depression. Nevertheless, they also observed that high levels of emotional clarity, when unaccompanied by emotional regulation, can intensify the negative emotional impact cybervictimization has on victims. Moreover, Extremera et al. [20] found that the development of EI components can reduce adolescent cybervictims’ risk of developing problems related to emotional adjustment as a consequence of cyberbullying. These authors suggest that high levels of EI may protect cybervictims’ well-being.

### The Present Study

According to the studies reviewed, despite the considerable attention attached to the relationship between EI and cybervictimization in the literature [17], there are still deficiencies in this aspect, especially regarding the role of EI in the emotional impact of cybervictimization. That is, there are no studies that analyze whether EI could have a positive, and possibly, compensatory influence on life satisfaction in the cybervictimization context, by preserving a positive self-concept and emotional stability (no depressive symptomatology).

In addition, cybervictimization has been associated with numerous emotional problems, as well as feelings of hopelessness and discomfort in the victims [13], but there are no studies that explore possible relationships between cybervictimization and the dimensions of EI that may clarify the influence that cybervictimization has on adolescents’ emotional adjustment. This study proposes moving in this direction to fill the gaps in these issues. Thus, the objectives of the present study were: (1) To analyze the relationship between cybervictimization and the emotional adjustment constructs (self-concept, depression, and life satisfaction); (2) to study whether EI as a whole can have a positive influence on self-concept, depression, and life satisfaction in the context of cybervictimization; (3) to examine the influence of each dimension of EI (emotional attention, clarity, and regulation) in the emotional impact of cybervictimization. Based on the previously reviewed research on cybervictimization in adolescence, the following hypotheses were established:

**Hypothesis** **1** **(H1).** *Cybervictimization will show a negative relationship with self-concept and life satisfaction, and a positive relationship with depression.*

**Hypothesis** **2** **(H2).** *Emotional intelligence will influence the relationship of cybervictimization with self-concept and depression, affecting, in turn, the relationship between each one of these variables with life satisfaction in the context of cybervictimization.*

**Hypothesis** **3** **(H3).** *The dimensions of EI (emotional attention, clarity, and regulation) will influence the relationship between cybervictimization and emotional adjustment.*

## 2. Materials and Methods

### 2.1. Ethics Approval

This study was approved by the Ethics Committee of the Miguel Hernández University (Ref.: PSI2015-65683-P), besides complying with the ethical values required for research with human beings and respecting the basic principles included in the Helsinki Declaration.

### 2.2. Participants

The participants in the study were 1318 adolescents (47% boys and 53% girls), aged between 11 and 18 years (*M* = 13.8, *SD* = 1.32), and enrolled in four Secondary Compulsory Education (SCE) schools in the Andalusian, Aragonese, and Valencian communities. Student distribution by academic grade was balanced: 24.7% were enrolled in the 1st grade of SCE, 27.3% in the 2nd grade, 23.7% in the 3rd grade, and 24.3% in the 4th grade. Sample selection was performed with probabilistic sampling, using as primary sampling units the urban geographical areas of the provinces of Alicante, Valencia, Seville, and Teruel, and as secondary units, the public schools in each area. The grades or classrooms were not used as tertiary units, as all the students of the four courses of SCE in all the schools participated. The schools were located in areas with a medium socioeconomic level. Approximately one-fourth of the parents of the participating students had primary education, four had secondary education, four had high school studies, and four had university studies. Most of the parents had paid employment outside the home: 86.7% of the fathers and 69.5% of the mothers.

### 2.3. Instruments

Cybervictimization. Victimization in cyberspace was measured using the Cybervictimization Scale (CYBVIC-R) [28]. This scale consists of 24 items that measure the harassment suffered by mobile phone and Internet during the last year: Harassment (e.g., “I have been insulted or ridiculed by someone on social networks or in groups like WhatsApp to really hurt me”), Persecution (e.g., “They have forced me with threats to do things I did not want to do on the Internet or the mobile”), Denigration (e.g., “They have told my secrets or revealed personal things about me without my permission on social networks or in groups”), Violation of Intimacy (e.g., “I have been recorded, or they have taken humiliating photos of me without my permission and they have distributed them on social networks”), Social Exclusion (e.g., “I have been ignored, and they have not answered my messages or things that I have sent to groups or social networks to make me feel bad “), Identity Theft (e.g., “They have used my profile or my accounts without me being able to prevent it”). The response scale ranges from 1 (never) to 5 (very often). The scale also has three items about the time and frequency of the aggressions and the perpetrator. The Cronbach alpha obtained in this work was 0.95.

Self-concept. The five dimensions of self-concept were measured using the Self-Concept Form-5 Scale (AF5) [29]. This 24-item scale measures four dimensions of self-concept (six items per dimension): Academic (e.g., “I work a lot in class”), Social (e.g., “I have trouble talking to strangers”), Family (e.g., “I am very happy at home”), and Physical (e.g., “I take care of myself”). The response scale ranges between 1 (strongly disagree) and 99 (strongly agree). The Cronbach alpha in the present study was 0.89 for the global scale (Academic 0.90; Social 0.76; Family 0.86, and Physical 0.79).

Depression was measured using the Depression Scale of the Center of Epidemiological Studies of the United States (CES-D) adapted to Spanish by Herrero and Meneses [30]. This 22-item instrument assesses from 1 (Never or very rarely) to 4 (Always or most times) the presence of depressive symptomatology in the last month. It provides a general index of depressed mood, assessing the symptomatology that usually accompanies it (e.g., “During the past month, I felt sad”). Cronbach’s alpha in this study was 0.84.

Satisfaction with Life. Life satisfaction was measured using the Satisfaction with Life Scale [31] adapted to Spanish by Atienza, Pons, Balaguer, and García-Merita [32]. This instrument contains five items that provide a general index of subjective perceived well-being (e.g., “I’m not happy with my life”). The items are rated on a four-point Likert-type scale ranging from 1 (strongly disagree) to 5 (strongly agree). In this study, the Cronbach alpha was 0.78.

Emotional intelligence. EI was measured using the Perceived Emotional Intelligence Scale (TMMS) [33] adapted to Spanish by Domínguez, Martín-Albo, Núñez, and León [34]. This scale consists of 22 items with five Likert-type response options from 1 (strongly disagree) to 5 (strongly agree), which provide a measure of EI based on three dimensions: Emotional Attention (e.g., “I think about my mood constantly”), Emotional Clarity (e.g., “Often, I am mistaken about my feelings”), and Emotion Regulation (e.g., “Although I sometimes feel sad, I have an optimistic viewpoint”). The global Cronbach alpha of this study was 0.91, and for the dimensions, it was 0.89, 0.86, and 0.87, respectively.

### 2.4. Procedure

The data of this research were collected as part of a larger study on psychological adjustment in adolescence in Spain. First, a letter with a summary of the research project was sent to the selected schools as a first step. Subsequently, an initial telephone contact with the school headmasters was established, followed by a meeting with all the teaching staff in which the objectives of the study and the procedure to be followed for data collection were reported. After the staff had agreed to participate, an explanatory letter was sent to the parents, requesting them to indicate in writing if they did not wish their child to participate (1% of parents used this option). The administration of the instruments was carried out by a group of trained and expert researchers in each region. Before data collection, students also attended a short briefing in which they provided written consent (none of the adolescents refused to participate). On the dates scheduled with the teaching staff, students were approached in their school classrooms to fill out the questionnaires voluntarily during a regular class period. The researchers of the present study administered the tools in the presence of the student’s tutor. The order of administration of the instruments was counterbalanced in each classroom and school. The instructions were read aloud, emphasizing the importance of answering all the questions and the anonymity of the answers. During the administration of the tests, the researchers were present to resolve doubts and ensure an unbiased process. Students could refuse to answer if they found it difficult to do so. The surveys that were suspicious in terms of the response patterns were not coded in the database (these surveys represented 1% of the total original samples).

### 2.5. Statistical Analysis

The original data matrix used for this study contained 1318 participants, with cybervictimization being the only construct that slightly exceeded a 10% of missing values, with items between 141 and 149 students without one or more values. A Missing Value Analysis (MVA) and a test of means showed that the average on the Cybervictimization Scale for participants with some missing value was significantly higher than for those who had answered all the questions. For this reason, it was decided to perform an automatic multiple imputation procedure using IBM SPSS 23 (IBM Corp., Armonk, NY, USA), through multinomial logistic regression models applied to each item of cybervictimization (values 1–5), considering each of the responded items as predictors, and each of the missing items as dependent variables, and selecting the most probable value. The number of cases that were finally imputed was only 58 because no imputation was made when a student refused to answer all the items on the Cybervictimization Scale. The final sample in this study was 1227 students.

The hypotheses were tested using structural equation methods. All estimates were made through a robust method because the data did not meet the multivariate normality criterion. The univariate distributions were non-normal, although the greatest deviation from normality was found in cybervictimization, where the high kurtosis indicated that the values were concentrated in a small range of the scale (Table 1). Mardia’s coefficient yielded a value of 443.23 for multivariate kurtosis and a value of 199,870.84 for multivariate skewness, both with *p* < 0.01, indicating non-normality [35]. Due to this evidence, the unweighted least squares (ULSs) method was applied to take deviations into account, because this method seems to produce more accurate estimates than other methods used to model non-normal variables [36]. Furthermore, considering the ordinal nature of the items in the measurement model, polychoric correlations were used [37].

The criteria used to compare the suitability of the proposed models were established following the combination of fit indixes and cutoff values recommended by Hu and Bentler [38,39]: Chi-square over degrees of freedom ratio with a recommended cutoff < 3; goodness-of-fit index (GFI), Tucker Lewis Index (TLI or NNFI), and comparative fit index (CFI) with recommended values > 0.95; standardized root mean square residual (SRMR) with a value < 0.08; a cutoff value close to 0.06 for the root mean square error of approximation (RMSEA), and expected cross-validation index (ECVI), for which smaller values indicate more potential for replication [40].

The analyses were performed using SPSS 23, and the package Lavaan 0.6.5 [41] in R 3.6.1 (R Foundation for Statistical Computing, Vienna, Austria), which allowed the estimation of the structural models using polychoric correlations and robust goodness-of-fit indices, including correction for the departure from multivariate normality [42,43].

## 3. Results

Half of the participants (50.5%) claimed to have experienced some of the types of indirect cybervictimization collected using the Cybervictimization Scale at least once during the academic year, while 52.7% declared to have been the victim of direct harassment. Among those who had been cybervictims, 77.3% suffered both direct and indirect cyberaggression. By far, the most frequent form of indirect cybervictimization was receiving calls to the mobile without obtaining an answer, just to annoy or scare (38.9%). Direct cybervictimization manifested in a much more varied way, such as receiving insults on social networks (27.9%), being a target of mocking comments, photos or videos uploaded online (26.4%), or being a victim of the disclosure of personal secrets in social networks or groups, such as WhatsApp or Snapchat (25.3%), among others.

Concerning the distribution and form of the variables considered in the study, Table 1 shows the summary of the descriptive statistics and correlations of the variables.

### Structural Models

To test the hypotheses of the study, three models were designed. Before building Model 1, two partial models were designed to ensure that there was empirical support for some theoretical relationships. The simplest one included only the direct relationship of cybervictimization with life satisfaction, showing a significant regression coefficient of −0.31 (*p* < 0.01). Cybervictimization had a significant negative coefficient but a small *R*^2^ (0.10), providing a scant explanation of the influence that it could have on life satisfaction in the absence of other variables of emotional adjustment on which it also has a theoretical influence. The second exploratory model was developed to test a possible influential effect of depression on the relationship between cybervictimization and life satisfaction. The result revealed that, when introducing the depression construct, the relationship of cybervictimization with life satisfaction shifted to almost null (−0.08), while presenting a significant coefficient of 0.32 with depression (*p* < 0.01), and revealing a significant negative relationship of −0.70 between depression and life satisfaction (*p* < 0.01). The explained variance of life satisfaction also increased (*R*^2^ = 0.54).

Based on the previous results, Model 1 of this study included self-concept acting together with depression as influencing variables in life satisfaction, themselves influenced by cybervictimization. EI was added in Model 2 to estimate its influence on previously observed relationships. Model 3 replaced the EI construct with the three dimensions that constitute it, to estimate the possible influence of each dimension on previously observed relationships.

**Figure 1:**
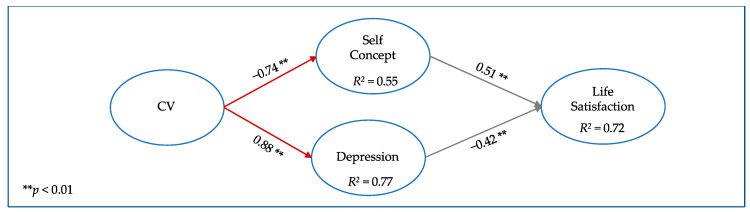
Model 1: Relationships between cybervictimization (CV) and emotional adjustment constructs.

**Figure 2:**
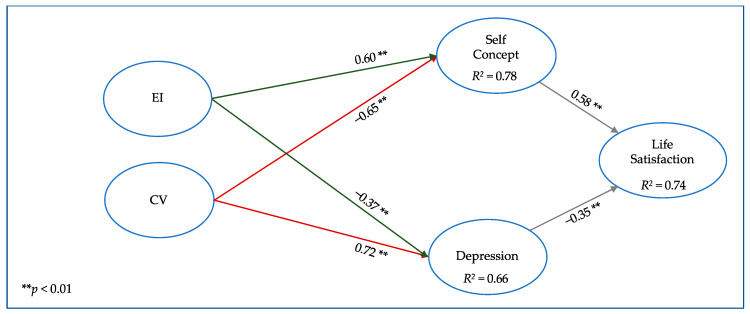
Model 2: Relationships between cybervictimization (CV) and emotional intelligence (EI) with emotional adjustment constructs.

**Figure 3:**
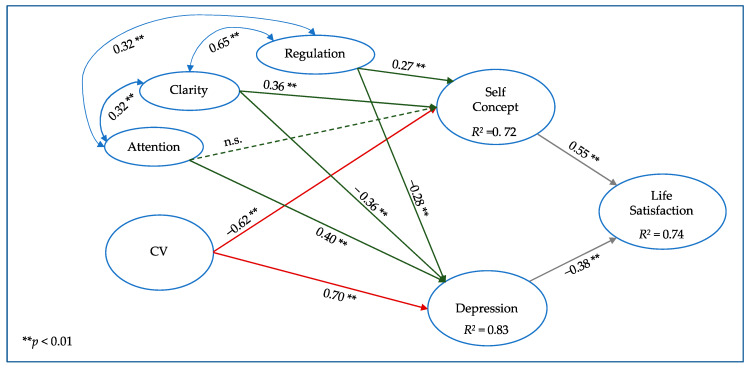
Model 3: Relationships between cybervictimization (CV) and the three components of emotional intelligence (EI) with emotional adjustment constructs. n.s.: non-significant coefficient.

Table 2 shows the robust goodness-of-fit indices of Models 1 to 3. Figure 1, Figure 2 and Figure 3 include the graphic models, the standardized regression coefficients, and the explained variance of the endogenous latent variables. For ease of viewing, the observed items and error terms have been omitted from the figures.

The first row in Table 2 indicates that Model 1 (Figure 1), which included cybervictimization and the constructs linked with emotional adjustment for this study, presents very good goodness-of-fit indices in general. This model is the basis for comparing the relationship of cybervictimization with emotional adjustment before and after considering IE. It confirmed the theoretical relationship of cybervictimization with the emotional factors, positively related to depression (0.88) and negatively to self-concept (−0.74). Moreover, as expected, the regression coefficient between self-concept and life satisfaction was positive (0.51), indicating that a high self-concept tended to be associated with high life satisfaction, whereas the relationship between depression and life satisfaction had a negative coefficient (−0.42).

The second model incorporated EI (Figure 2) and showed a very similar goodness-of-fit to Model 1, with only SRMR being slightly higher than in the previous model and all the indices within the predetermined limits. In this model, cybervictimization remained negatively associated with self-concept (−0.65) and positively with depression (0.72). EI had a positive coefficient (0.60) with self-concept and a negative one with depression (−0.37), both with *p* < 0.01, which implies a positive impact, contrary to the influence of cybervictimization on emotional adjustment variables.

In Model 3 (Figure 3), emotional attention, emotional clarity, and emotion regulation were included as independent constructs. Clarity and regulation showed a high covariance with each other (0.65), whereas attention had a lower relationship with both of them (0.32). This model had similar goodness-of-fit indices to the previous models in Table 2 but revealed the smallest estimation errors, with the lowest RMSEA and SRMR indices. It also produced higher *R*^2^ coefficients in all the endogenous variables (self-concept, depression, and life satisfaction) compared with Model 1, thus indicating its explanatory superiority.

In global terms, Figure 3 shows that cybervictimization and emotional attention, the latter considered now independently, had a similar pattern, being negatively associated with self-concept and positively with depression. The other dimensions of EI, emotional clarity and regulation, showed the opposite relationship with the variables of emotional adjustment.

Now considering the factors related to EI, the influence of emotional attention on self-concept was nonsignificant, but it was very significant on depression (0.40), indicating that this EI dimension itself is associated with depressive symptoms.

Emotional clarity showed a positive relationship with self-concept (0.36) and a negative one with depressive symptoms (−0.36). Emotion regulation, the other dimension of EI, presented the same pattern as emotional clarity: a positive relationship with self-concept (0.27) and a negative one with depression (−0.28).

Considering that self-concept showed a positive relationship with life satisfaction and that depression presented a negative relationship, we could expect to find students with a higher life satisfaction if they show high levels of EI, specifically emotional clarity and regulation, in the context of cyberbullying.

## 4. Discussion

The objective of the present study was to analyze the relationship between EI and self-concept, depression, and life satisfaction, as well as the influence of the EI dimensions on emotional adjustment in the context of cybervictimization. The results indicated that EI can influence the relationship between cybervictimization and emotional variables, and also, that each of the EI dimensions plays an important role in this relationship, although not all in the hypothesized direction.

The first hypothesis proposed in this study postulated that cybervictimization would have a negative relationship with self-concept and life satisfaction, and a positive relationship with depression. The results confirm this hypothesis because cybervictimization had a negative relationship with self-concept and a positive relationship with depression. In this sense, cybervictimization was indirectly related to life satisfaction through self-concept and depression. This result coincides with previous works suggesting that cybervictimization is associated with several consequences that impact on victims’ emotional adjustment, such as higher levels of depressive symptomatology [6,7] and negative self-concept [9,10]. In addition, self-concept seems to play an important role in adolescents’ life satisfaction, as it is considered one of the main factors related to their well-being [44]. Regarding the relationship between depression and life satisfaction, studies with adolescent samples indicate that these two variables are strongly and negatively correlated [45].

Regarding the second hypothesis, it stated that EI would influence the relationship of cybervictimization with self-concept and depression, affecting the relationship between each one of these variables and life satisfaction in the context of cybervictimization. The results obtained allow us to confirm the hypothesis. EI showed a strong positive relationship with self-concept and a high negative relationship with depression. Moreover, by introducing EI, cybervictimization remained negatively related to self-concept and positively to depression, but the influence of these relationships was less than in the previous model. These results pointed to a possible suppressive influence of EI on cybervictimization concerning emotional adjustment. In line with previous studies of EI and cyberbullying, high levels of EI attenuated the negative influence of cyberbullying on emotional problems [46]. In addition, other researchers suggest that the emotional skills of EI, that is, perceiving emotions, understanding the causes and consequences of emotions, and regulating one’s emotions and those of others might reduce the risk of experiencing psychosocial problems as a consequence of cybervictimization [17]. Thus, results revealed the importance of EI in the relationship between cybervictimization and emotional adjustment. In line with the influence of EI in life satisfaction, understanding and managing emotions appropriately could be related to well-being and life satisfaction by reducing the risk of psychosocial maladjustment [47]. Previous studies confirm that students’ higher levels of EI make it easier for them to identify, understand, and regulate their negative emotions in the face of stressful events, and to maintain positive moods when situations require it, contributing to their satisfaction with life [48]. Although research on cybervictimization was related to life satisfaction, the influences of EI on self-concept and depressive symptomatology were not simultaneously considered in this relationship until now. The main contribution of the present study in this sense is that, in this work, the dimensions of EI and other emotional variables were analyzed in relation to life satisfaction in the context of cybervictimization.

The third hypothesis of this study stated that the three components of EI would influence the relationship between cybervictimization and emotional adjustment. In line with this hypothesis, the relationship between cybervictimization and emotional adjustment was influenced by the presence of EI dimensions. Thus, emotional clarity and regulation influenced the relationships between cybervictimization and self-concept, and between cybervictimization and depression. Specifically, emotional clarity and regulation had a negative direct influence on depression, and a positive direct influence on self-concept and life satisfaction. Consistent with previous literature, high scores in emotional clarity and regulation are inversely related to depression [15], but positively related to levels of well-being and life satisfaction [49,50]. However, in contrast to the third hypothesis, emotional attention affected the symptoms of depression, and no direct relationship between emotional attention and self-concept was observed. As emotional attention is related to perceiving one’s own emotions, too much attention with a low level in the other EI dimensions may lead to weak emotional control and affect emotional adjustment [15]. Thus, and as previously mentioned, the EI dimensions have a total positive influence on the problems associated with cybervictimization due to emotional clarity and regulation, but in the opposite direction, as emotional attention per se can affect that relationship negatively. In any case, results revealed the importance of each EI dimension in the relationship between cybervictimization and emotional adjustment.

### Imitations and Future Research

Although this study has some significant strengths, such as the analysis of the relationship between cybervictimization and life satisfaction, as well as the study of EI’s role in the association between cybervictimization and emotional adjustment, it also presents several limitations to be acknowledged for further research. A first limitation is based on the cross-sectional nature of the data, making it impossible to establish causal relationships between the analyzed variables. A longitudinal study of measures at different times would provide further insights into the causal relationships among study variables. Secondly, it must be considered that, although questionnaires were administered anonymously, self-administered instruments in adolescence could generate response bias, affecting the validity and generalization of the data. The complementarity of the study findings with data from other informants (parents, teachers, or peers) could lead to a broader and more comprehensive approach to the topic. Further, it must be stressed that the results of this study are limited to the adolescent stage from 11 to 17 years, so they are not generalizable to subjects of other ages or other educational levels (early childhood education, primary education, and higher education), or even to school settings belonging to other cultures. Finally, the results show the need to further analyze the relationship between emotional attention and emotional maladjustment with and without the presence of high levels of clarity and emotion regulation. Relatedly, future studies should examine the potential influence of EI considering samples of children and adolescents, as well as specific samples of cybervictims.

## 5. Conclusions

In conclusion, the results of this study expand the knowledge about the influence of cybervictimization in adolescents’ emotional adjustment. Cybervictimization is associated with several negative consequences, some which might explain why victims of cyberbullying report lower satisfaction with life. Regarding EI, the findings of the present study allow us to conclude that the development of emotional skills influences the relationship between cybervictimization and emotional problems. In this sense, EI, considered as a multidimensional concept, might also be considered as a personal resource to mitigate the emotional maladjustment related to cybervictimization. Accordingly, this study highlights the importance of EI to influence psychosocial problems, especially associated with cyberbullying behavior. Moreover, concerning the theoretical contribution of this study to the literature, its findings could also be used to develop cyberbullying prevention programs integrated in scholar centers, aimed at increasing adolescents’ emotional skills to protect them against, or at least mitigate, the negative consequences of being a victim of cyberbullying.

## Figures and Tables

**Table 1:** Descriptive statistics and Spearman correlations for the variables included in the study.

Variables	1	2	3	4	5	6	7	8
1 Cybervictimization (1–5)	1.00							
2 Emotional Intelligence (1–5)	−0.07 *	1.00						
3 EI: Emotional Attention (1–5)	0.08 **	0.71 ** (a)	1.00					
4 EI: Emotional Clarity (1–5)	−0.12 **	0.80 ** (a)	−0.35 **	1.00				
5 EI: Emotional Regulation (1–5)	−0.14 **	0.79 **(a)	−0.33 **	0.53 **	1.00			
6 Self-Concept (0–9)	−0.22 **	0.37 **	0.13 **	0.39 **	0.34 **	1.00		
7 Depression (1–4)	0.36 **	−0.20 **	0.11 **	−0.30 **	−0.27 **	−0.43 **	1.00	
8 Life Satisfaction (1–5)	−0.28 **	0.30 **	0.05	0.35 **	0.33 **	0.55 **	−0.52 **	1.00
Statistics								
Mean	1.34	3.32	3.25	3.33	3.38	6.42	1.86	3.73
SD	0.62	0.71	0.96	0.83	0.94	1.31	0.65	0.86
Skewness	2.67	−0.08 *	−0.64 *	−0.04 *	−0.19 *	−0.74 *	0.82	−0.47 *
Kurtosis	6.73	0.04	−0.56 *	−0.32 *	−0.57 *	0.34	−0.22 *	−0.49 *

Notes: *: *p* < 0.05; **: *p* < 0.01; (a): Considered observed variables for the Emotional Intelligence construct in Model 2, but introduced independently as constructs in Model 3, which did not include Emotional Intelligence.

**Table 2:** Models fit indicators.

Models	*X* ^2^_S-B_	*df*	GFI	CFI	TLI	RMSEA	SRMR	ECVI
Model 1	2840.78	508	0.99	0.98	0.98	0.046 (0.044; 0.047) *	0.050	1.51
Model 2	3557.85	608	0.99	0.98	0.98	0.047 (0.046; 0.049) *	0.054	1.88
Model 3	6359.42	1415	0.99	0.98	0.97	0.044 (0.043; 0.045) *	0.049	3.98

Note: * Confidence Interval 90%. X^2^_S-B_: Satorra-Bentler adjusted *chi-square*; *df*: degrees of freedom; GFI: Goodness of Fit statistic; CFI: Comparative Fit index; TLI: Tucker Lewis index; RMSEA: Root Mean Square Error of approximation; SRMR: Standardized Root Mean Square Residual; ECVI: Expected Cross-validation Index.
